# Primary Intramedullary Spinal Melanocytomas: Case Report and Review of Clinical Features, Diagnosis, and Management

**DOI:** 10.3390/jcm14228047

**Published:** 2025-11-13

**Authors:** Gil Kimchi, Samantha Varela, Juan Pablo Zuluaga-Garcia, Francisco Call-Orellana, Esteban Ramirez Ferrer, Romulo Augusto Andrade de Almeida, Maria A. Gubbiotti, Isabella C. Glitza, Andrew J. Bishop, Jonathan D. Grant, Robert Y. North, Christopher A. Alvarez-Breckenridge, Laurence D. Rhines, Claudio E. Tatsui

**Affiliations:** 1 Department of Neurosurgery, University of Texas MD Anderson Cancer Center, Houston, TX 77030, USA; jpzuluaga@mdanderson.org (J.P.Z.-G.); facall@mdanderson.org (F.C.-O.); eramirez14@mdanderson.org (E.R.F.); randrade@mdanderson.org (R.A.A.d.A.); rnorth@mdanderson.org (R.Y.N.); calvarez11@mdanderson.org (C.A.A.-B.); lrhines@mdanderson.org (L.D.R.); cetatsui@mdanderson.org (C.E.T.); 2 Department of Neurosurgery, Baylor College of Medicine, Houston, TX 77030, USA; samantha.varela@bcm.edu; 3 Department of Pathology, University of Texas MD Anderson Cancer Center, Houston, TX 77030, USA; magubbiotti@mdanderson.org; 4 Department of Melanoma Medical Oncology, University of Texas MD Anderson Cancer Center, Houston, TX 77030, USA; icglitza@mdanderson.org; 5 Department of Radiation Oncology, University of Texas MD Anderson Cancer Center, Houston, TX 77030, USA; abishop2@mdanderson.org; 6 Department of Radiation Oncology, Intermountain Health, Murray, UT 84107, USA; jon.grant@imail.org

**Keywords:** melanocytoma, melanocytic tumor, intramedullary tumor, spine oncology, intradural tumor

## Abstract

**Objective:** Intramedullary melanocytomas are extremely rare spinal cord tumors with distinct histopathological and imaging characteristics. This report reviews the literature on this pathology and presents a representative case study, highlighting aspects of diagnosis and management. **Methods:** A scoping review of PubMed, Web of Science, and Embase databases was conducted to identify reports on intramedullary melanocytomas, focusing on clinical presentation, imaging features, histopathology, treatment, and outcomes. Case reports and case series were included due to the rarity of these tumors. **Results:** Twelve manuscripts met the inclusion criteria, including 15 patients. In the majority of patients, intramedullary melanocytomas present with progressive myelopathy and pain. Most common MRI findings include hyperintensity on T1-weighted images, iso- to hypointensity on T2-weighted images, and homogeneous contrast enhancement. Intralesional cysts and associated syrinx are common. Gross total resection (GTR) remains the primary treatment, but complete removal is often challenging due to tumor adherence to neural structures. **Conclusions:** Intramedullary melanocytomas require careful diagnosis and management due to their diagnostic overlap with malignant melanoma and potential for recurrence. While GTR is the mainstay of treatment, long-term surveillance is warranted due to high recurrence rates. Further research is needed to define the natural history of the disease and establish optimal therapeutic strategies.

## 1. Introduction

Melanocytic tumors of the central nervous system (CNS) are rare lesions arising from leptomeningeal melanocytes. Embryologically, these pigmented cells arise from melanoblasts of the neural crest which then migrate to peripheral sites. Therefore, physiologically melanocytic cells are primarily located in the upper cervical spine and skull base. CNS melanocytic tumors represent a minute fraction of CNS neoplasms and only <1% of all melanomas [1,2]. Melanocytic neoplasms typically present in a localized or diffuse fashion, with diffuse lesions primarily seen in dermatological syndromes such as nevus of Ota and neurocutaneous melanosis [3]. Given their embryological origins, most CNS melanocytic tumors occur in the posterior fossa and cervical spine; however, caudal spinal involvement has been reported [4].

Central nervous system melanocytic tumors exist along a spectrum from benign melanocytomas to malignant melanomas, with intermediate forms exhibiting variable biological behavior. Benign meningeal melanocytomas are generally localized, circumscribed lesions which do not invade surrounding tissues, melanocytic lesions which exhibit parenchymal invasion or cellular atypia are classified as intermediate grade lesions, while malignant melanomas are aggressive neoplasms which unequivocally invade parenchymal structures and result in local necrosis [2]. In contrast to cutaneous melanomas, meningeal melanocytomas are generally localized lesions which do not exhibit systemic disease. Diffuse meningeal melanocytic tumors exist, however, these are generally associated with congenital neurological disorders such as neurocutaneous melanosis, which is associated with diffuse meningeal melanocytic lesions and cutaneous findings, and nevus of Ota (blue nevus melanoma) a condition with trigeminal and ocular nerve involvement which results in ocular hyperpigmentation [2]. Interestingly, CNS melanocytic tumors are also molecularly distinguishable from cutaneous melanocytic lesions, as they primarily exhibit alterations in genes infrequently associated with cutaneous melanomas. CNS melanocytic tumors, including neurocutaneous melanosis and nevus of Ota, frequently exhibit mutations in the GNAQ and GNA11 genes, as compared to BRAF, NRAS, HRAS, and KIT mutations seen in non-CNS originating melanocytic lesions [3].

Primary CNS melanocytic lesions are extremely rare tumors which exhibit unique histopathological characteristics. Localized benign solitary melanocytic lesions are primarily intradural and extramedullary, given their leptomeningeal origin. Intramedullary melanocytic lesions are therefore exceptionally rare and distinct from their more common extramedullary counterparts—with an estimated incidence of roughly 1 per 10 million people annually [2]. Even though both extramedullary and intramedullary melanocytomas are generally considered low-grade tumors, their clinical course is highly variable. Similar to other intramedullary primary CNS tumors, intramedullary melanocytomas typically present with progressive myelopathy due to direct compression or local infiltration of spinal cord structures. While these lesions are generally slow-growing, reports of recurrence, malignant transformation, and even metastatic spread suggest a more complex biological potential [1].

Given the rarity of intramedullary melanocytomas, there is limited literature describing their natural history, optimal management strategies, and long-term outcomes. This review focuses specifically on intramedullary melanocytomas, summarizing the current literature on their clinical presentation, diagnostic challenges, histopathological characteristics, treatment approaches, and prognosis. Additionally, we describe a representative case to illustrate key aspects of the clinical course and management considerations for this rare tumor.

## 2. Methods

### 2.1. Study Design

This review aimed to analyze the existing literature on intramedullary melanocytomas. Because this entity is exceptionally rare, we included single-patient case reports and case series to capture all available clinical data. The review followed PRISMA 2020 guidance. Ethical approval was not required.

### 2.2. Inclusion and Exclusion Criteria

Eligible designs were retrospective or prospective case series, cohort studies, and case reports that reported primary intramedullary melanocytoma confirmed by histopathology and/or immunohistochemistry. Studies limited to intracranial melanocytomas, extramedullary spinal melanocytomas, non-melanocytic pigmented tumors (e.g., melanotic schwannoma), or metastatic melanoma were excluded. We also excluded non-English articles, reviews, editorials, comments, and conference abstracts.

### 2.3. Literature Search Strategy

A targeted search was conducted in PubMed, Web of Science, and Embase to identify studies published until February 2025. The search strategy incorporated a combination of free-text keywords and controlled vocabulary, including terms such as “intramedullary melanocytoma”, “spinal melanocytic tumor”, and “primary spinal melanoma.” Boolean operators (AND, OR) were used to refine the search results.

### 2.4. Study Selection

**Figure 1:**
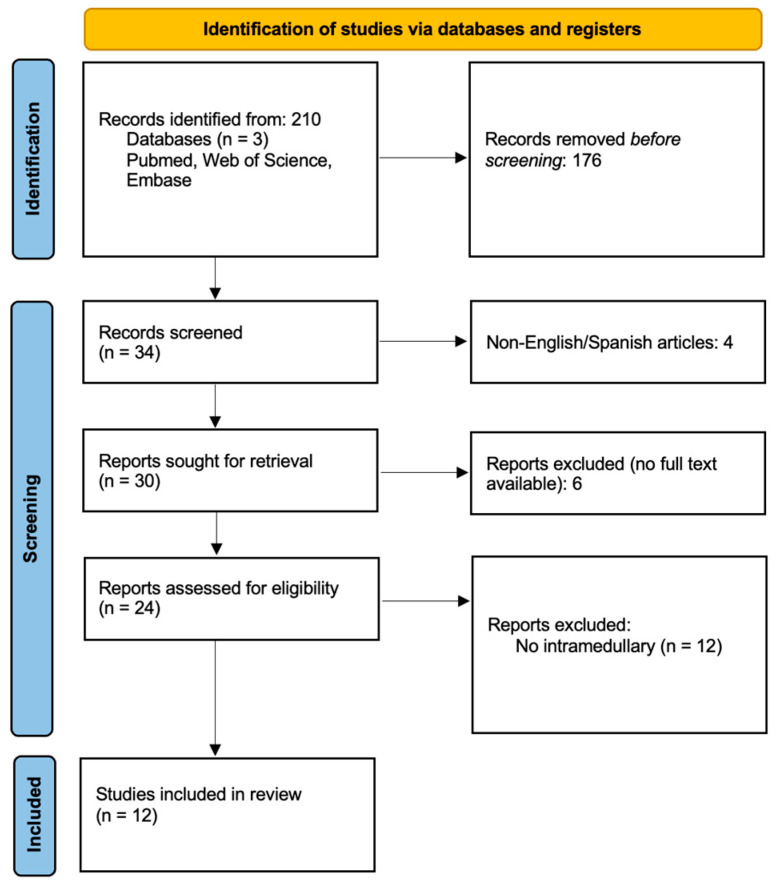
PRISMA flowchart.

All retrieved citations underwent a structured selection process. After removing duplicates, an initial review of titles and abstracts was conducted to assess relevance. Full-text articles meeting the inclusion criteria were further evaluated for eligibility. Two independent reviewers conducted the initial screening of titles and abstracts and a third reviewer resolved any disagreements. Figure 1 displays the PRISMA flowchart for the identification and screening of relevant publications.

### 2.5. Data Extraction and Analysis

Data were systematically extracted using a standardized template, capturing the study design, patient demographics, tumor location, imaging characteristics, histopathological findings, treatment approaches, recurrence rates, and malignant progression. Standard descriptive statistics were used for quantitative findings, while qualitative analysis was conducted to identify recurring themes and trends in diagnosis and management. Study selection and reasons for exclusion were documented per PRISMA 2020. Figure 1 presents the PRISMA flow diagram detailing records identified, screened, reviewed in full text, and included in the final synthesis.

## 3. Results

Overall, twelve reports were included, which yielded 15 patients. A flowchart summarizing the data selection process is presented in Figure 1. Table 1 provides a summary of reports describing the presentation and outcomes of primary intramedullary melanocytic tumors, with the largest case-series comprising three patients [5]. The majority of the cases within our cohort exhibited lesions within the thoracic spinal cord (n = 11, 73.3%), with the rest of the cases being in the cervical spinal cord (n = 4, 26.6%). The median age was 46 (IQR 19–79). All but three patients presented with motor deficits; however, all patients within the cohort presented with a neurological symptom. In most cases (n = 11, 73.3%), gross total resection (GTR) was achieved. Of the patients in whom GTR was achieved, four (36.3%) experienced recurrence, while both patients in the sub-total resection cohort experienced tumor recurrence. Most patients (n = 11, 73.3%), remained neurologically stable and improved following resection. MelanA positivity was reported in 7 patients, S100 positivity in 11 patients, and HMB-45 positivity in 8 patients, with the maximal Ki-67 reported in the cohort being 5%, however molecular markers were not present for all patients. Recurrence following surgery occurred in six patients (40%), but only two patients out of the cohort received post-operative radiation.

### Case Report

**Figure 2:**
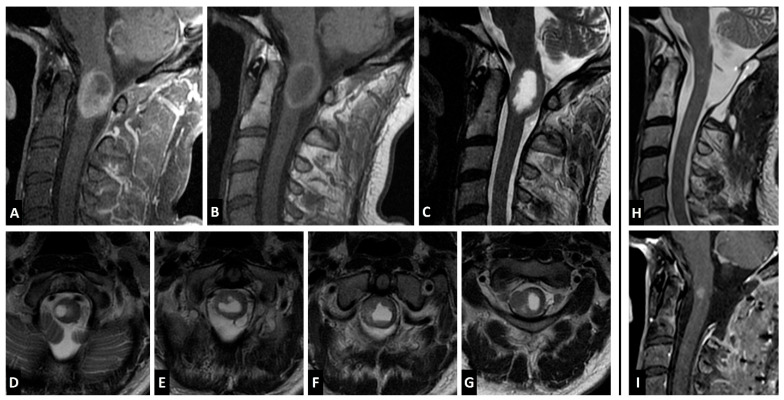
Pre- and postoperative MRI of a 44-year-old male with an intramedullary melanocytic tumor. (**A**) Midsagittal T1 post-Gd sequence. Note the vivid enhancement of the solid tumor component. (**B**) Non-Gd T1 weighted image. Note the hyperintense rim characteristic of melanin-rich pathologies. (**C**) T2-weighted midsagittal image, demonstrating a homogenous cystic core and the absence of cord edema or associated syrinx. (**D**–**G**) Note the lack of clear margin between the tumor and the cord parenchyma. (**H**,**I**) postoperative T2-weighted image (**H**) and post-Gd scan (**I**) demonstrating a small residual enhancing mass at the ventral aspect of the resection cavity.

**Figure 3:**
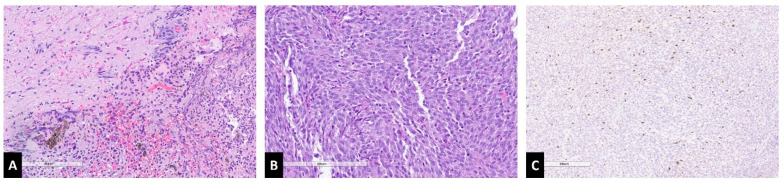
Histopathological slides. (**A**) Piloid gliosis with tumor. (**B**) Nuclear atypia. (**C**) Ki67 stain.

A 44-year-old man presented with suboccipital pain and mild cervical myelopathy, manifesting as decreased hand dexterity and gait imbalance. Despite gradual symptom progression, his deficits remained mild, and he continued to ambulate unassisted. Initial evaluation at an outside institution included a cervical spine MRI, which revealed an expansile cystic intramedullary lesion at the cervicomedullary junction. The non-cystic component was isointense to the spinal cord on T2-weighted images, and hyperintense on non-Gd T1 (Figure 2C–G). The solid component enhanced homogenously with gadolinium (Figure 2A). A total neuroaxis MRI was performed, confirming the absence of additional lesions. The patient underwent an open biopsy at an outside hospital, which revealed darkly pigmented tissue consistent with a melanocytic neoplasm. He remained clinically stable for one month, after which he experienced progression of the myelopathy. Follow-up MRI demonstrated a mild increase in lesion size, leading to referral for definitive surgical intervention. Systemic staging with CT of the chest, abdomen, and pelvis was performed and was negative for additional sites of disease. At our institution, given the patient was symptomatic with evidence of disease progression, the patient underwent tumor resection via a C1–2 laminectomy. He was positioned prone with the head secured in a Mayfield pinholder, and continuous intraoperative motor-evoked potentials (MEPs) and somatosensory-evoked potentials (SSEPs) were monitored. The prior laminectomy was expanded, and intraoperative ultrasound confirmed adequate exposure. Following dural opening, a dark, exophytic lesion was identified under the operative microscope. After pial incision, direct invasion of the tumor into the cord parenchyma was identified, and there was no overt plane between the lesion and the cord. During the final phase of the removal, an 80% decrease in SSEPs and 40% decrease in MEPs was observed, leading to the decision to leave a small adherent portion on the ventral aspect of the tumor, achieving near-total resection. Postoperatively, the patient exhibited transient worsening weakness, graded 4/5 in the right upper extremity and 4-/5 in the right lower extremity. Over the next month, he regained full strength on the left upper and lower extremities, with no sensory deficits and intact gait, albeit he did sustain a right 4/5 hand grip weakness and persistent spasticity in the right upper extremity and lower extremities bilaterally. Given the near total resection, the decision was made to treat with adjuvant radiation to the operative bed at C1-2 (45 Gy in 25 fractions, RapidArc technique with 6 MV photons). At 13-month follow-up, MRI demonstrated a stable residual enhancing lesion without evidence of progression. Pathological analysis confirmed leptomeningeal melanocytoma, and detection of the *GNAQ* mutation, in the absence of a history of uveal melanoma or blue nevus-like melanoma, this supported the diagnosis of a primary melanocytic lesion rather than a melanotic nerve sheath tumor (Figure 3).

## 4. Discussion

CNS melanocytomas are rare, slow-growing lesions which originate from the leptomeninges. Most central nervous system melanocytomas are focal, intradural extramedullary lesions, while intramedullary melanocytomas are incredibly rare and exhibit distinct pathological and clinical characteristics. The diagnosis of CNS melanocytomas relies on imaging, but primarily histopathology, given their radiographic similarities to malignant melanomas. Although they are generally considered low-grade lesions, their management remains challenging due to their invasiveness to the cord parenchyma and potential for recurrence. Herein, we review their clinical features, diagnosis, and management, summarizing the current literature and insights from our case.

### 4.1. Epidemiology

Melanocytomas are exceptionally uncommon, with an incidence rate of 1/10,000,000 [2], of these cases, the majority are extradural, with intradural melanocytomas representing only a paucity of cases documented in the literature as of 2024 [16]. Given their rarity, definitive incidence rates remain unknown, and most available data are derived from individual case reports and limited case series. These tumors have been identified across a wide age spectrum, ranging from pediatrics to adults; however, they are most prevalent among the fifth decade of life [2]. Meningeal melanocytomas are exhibit an even distribution between males and females, with near equal prevalence noted among males/females in our analysis. While melanocytic tumors of the spine more frequently arise in the extramedullary leptomeninges, intramedullary melanocytomas predominantly occur in the thoracic spinal cord. Prior studies report that 53% of intramedullary melanocytomas arise from the thoracic cord, followed by cervical (28%), and lastly the lumbosacral spine (19%). In the cohort of cases included in our study, 73% of intradural melanocytomas were within the thoracic spine, with the remaining cases being in the cervical spine.

### 4.2. Histopathology and Genetic Markers

At the macroscopic level, melanocytomas appear to be brown-reddish in appearance and are well demarcated [2]. Melanocytomas can be categorized as extradural and rarely intradural and can be further delineated into low versus intermediate grade tumors, as based on the WHO 2021 classification guidelines [2].

In general, melanocytomas are composed of heavily pigmented cells, typically arranged in compact nests, sheets, or fascicles [17]. Microscopically, low-grade melanocytomas are densely packed, have bean-shaped or oval nuclei with small eosinophilic nucleoli, they exhibit low mitotic activity (0–1 per 10 high-power fields), and have a low MIB-1 labeling index (<1–2%) [2]. Intermediate grade tumors exhibit increased mitotic activity (2–5 per 10 high-power fields) and invade the underlying tissue, they generally lack necrosis but may have cellular atypia. On immunohistochemical analysis, these tumors are positive for HMB-45, Melan-A, and S-100 protein, while lacking expression of epithelial membrane antigen (EMA), GFAP, and keratins, they also generally have low Ki-67 proliferation rates. In contrast, malignant melanomas display larger, atypical, spindled pigmented cells with higher mitotic activity (average 5.7 per 10 high-power fields), they frequently have irregular eosinophilic nuclei with numerous mitotic figures, and an elevated MIB-1 labeling index (mean, 8.1%) [16]. Malignant melanomas frequently show necrosis or CNS invasion and may contain pleomorphic, bizarre nuclei, reflecting their more aggressive nature.

Molecular genetic alterations between primary central nervous system melanocytic lesions and epithelial-derived melanocytic tumors vary significantly. Melanocytic lesions derived from the epithelium frequently display mutations or alterations in components of the MAP kinase pathway, these mutations are frequently harbored on exons within chromosome seven and one, which harbor the BRAF and NRAS genes [3]. These genes encode for a protein kinase (BRAF) and a GTP-binding protein (NRAS) responsible for activating the RAS and MAP kinase pathways. Up to 50% of cutaneous melanocytic tumors exhibit BRAF mutations, while 30% exhibit NRAS mutations. In contrast, CNS melanocytomas rarely convey BRAF and NRAS mutations, rather, they harbor mutations in the GNAQ and GNA11 genes which are located on chromosome nine. A total of 60–70% of CNS melanocytomas harbor mutations in GNAQ and GNA11, and do not harbor mutations in BRAF and NRAS [2]. GNAQ and GNA11 encode the GTP-binding proteins responsible for the activation of the MAP kinase pathway; therefore, mutations result in constitutively active MAP kinase signaling resulting in cell growth. Interestingly, uveal melanomas, or melanomas which originate from the choroid, as well as diffuse melanocytic CNS lesions, such as neurocutaneous melanosis and blue nevus melanoma/nevus of Ota also frequently exhibit GNAQ and GNA11 mutations. These genetic alterations are rarely observed in cutaneous or mucosal melanomas, suggesting that GNAQ and GNA11 may be involved in the early migration of melanocytes during embryogenesis, given GNAQ is important in melanocyte survival during early neural crest development [3]. Ultimately, both cutaneous and CNS melanocytic lesions exhibit alterations in the MAP kinase signaling pathway; however, given their distinct genetic alterations, targeted systemic treatment is varied.

Although melanocytomas are largely composed of highly pigmented cells, a small proportion may lack pigment, therefore they can further be distinguished from epithelial melanocytic lesions via fluorescent in situ hybridization (FISH). Melanocytomas typically demonstrate normal findings on four-color for chromosomes 3, 7, 17, and 9p21/*CDKN2A*, a key feature that differentiates them from metastatic melanomas [18,19,20,21].

### 4.3. Imaging Characteristics

Wagner et al. [9] reported that intramedullary melanocytomas typically appear iso- to hyperintense on T1-weighted MRI and hypointense on T2-weighted sequences, with homogeneous contrast enhancement. These signal characteristics are largely influenced by melanin content and the inert metal scavenging properties of melanin. Therefore, highly pigmented tumors with high melanin content (>10%) exhibit more pronounced T1 hyperintensity and T2 hypointensity, whereas less pigmented lesions may show more variable signal intensities [2]. Melanocytic lesions also generally show signal loss within susceptibility imaging (SWI), generally due to micro-hemorrhages within melanocytic lesions; however, micro-hemorrhages are typically features of chronic lesions (>3 months) and may not be present at symptom presentation [2]. Of the 16 cases included in our study (including our own case), 14 (87.5%) exhibited T1 hyperintensity and T2 hypo or iso intensity on MRI.

Most CNS melanocytic lesions appear as solitary lesions within the posterior fossa or cervical spine, with supratentorial lesions being extremely rare. Within the spinal canal, most lesions will appear intradural and extramedullary; however, both extra- and intra-medullary melanocytic tumors are generally well-circumscribed, they may appear as hemorrhagic lesions with focal nodular enhancement, and also, frequently have an associated syrinx. These lesions may also extend into the neuroforamen resulting in neuroforaminal enlargement and remodeling, which may mimic the appearance other primary CNS lesions, such as neurofibromas, meningiomas, or schwannomas [2]. In contrast, malignant melanomas tend to display more aggressive imaging features, including irregular borders, necrosis, and invasion into adjacent tissues, making the differentiation between the two based on MRI characteristics challenging [22]. Additionally, CT and radiographs are typically unhelpful, as these tumors rarely demonstrate calcifications or bone involvement; however, these lesions may appear hyperintense on CT images and may mimic acute hemorrhage. Given the significant overlap in imaging characteristics between melanocytomas and malignant melanoma, histopathological and molecular analysis remain essential for definitive diagnosis.

### 4.4. Clinical Presentation

The symptoms of intramedullary melanocytic tumors often mirror those of other intramedullary pathologies but generally follow a more indolent course. In most cases, pain is an early symptom, followed by either localized neurological or radicular symptoms, with many patients experiencing progressive debilitating myelopathy. Neurological deficits, including weakness, gait disturbances, and sensory changes, may develop gradually, though acute deterioration can occur due to hemorrhage or decompensation secondary to mass effects. Their presentation is nonspecific and often requires imaging for further evaluation. The cases reported in our study highlight these findings, given that 81% (n = 13) of patients experienced severe neurological symptoms including lower extremity paraparesis, bowel and bladder dysfunction, and significant paresthesia.

### 4.5. Treatment and Outcomes

Surgical resection remains the primary treatment for intramedullary melanocytomas. Specifically, gross total resection remains the gold standard, given that patients who undergo gross-total resection of their tumors rarely have disease recurrence [15]. Given their morphological appearance, the goal of surgery is similar to other dural based lesions, with the goal being the decompression of neural structures and complete tumor removal with removal of the diseased dura. Although favorable surgical outcomes have been described, gross total resection is challenging to achieve in these cases, given that intramedullary melanocytic tumors frequently adhere to surrounding structures and are densely adhered to the dura, increasing the risk for neurological compromise. In our cohort, gross total resection was achieved in 11 patients, four of these had tumor recurrence (36%), while two of the four cases (50%) with near-total or sub-total resection experienced tumor recurrence. Our experience highlights the technical challenges associated with these lesions, the tumor was directly invading the underlying parenchyma, with no clear plane during the final phase of removal, a decrease in SSEPs/MEPs was noted; therefore, resection was stopped and near-total resection was achieved, however, to date the patient has not experienced disease recurrence or growth.

Given the rarity of these lesions, post-operative radiation to the tumor bed has not been standardized among practice. While stereotactic radiation has been utilized for intracranial melanocytomas [23], it is not a feasible primary treatment for symptomatic intramedullary spinal melanocytomas. Given their low incidence, radiotherapy data from cutaneous metastatic melanoma involving the CNS has been used to treat melanocytic lesions, with conventional fractionated radiation (60 Gy) delivered to tumors with sub-total resection and stereotactic radiosurgery delivered in patients with small residual lesions [2]. In our cohort, three patients (including our case) received post-operative radiation, our patient did not experience recurrence; however, tumor recurrence was appreciated in the two other patients.

## 5. Conclusions

Central nervous system melanocytomas are rare, circumscribed, slow-growing lesions which arise from leptomeningeal melanocytes. Given their embryological origins, CNS melanocytomas frequently arise from the upper cervical spine and skull base but can present in the distal spinal cord. CNS melanocytomas harbor distinct histological and genetic alterations when compared to their cutaneous and mucosal counterparts. The majority of CNS melanocytic spinal lesions are intradural and extramedullary with only a paucity of cases being intramedullary. Intramedullary melanocytomas require careful diagnosis and management due to their potential for recurrence and diagnostic overlap with malignant melanoma. While surgery is the mainstay of treatment, long-term surveillance is essential. Further research is needed to define optimal therapeutic strategies.

## Figures and Tables

**Table 1:** Summary of the included studies.

Study Author	Year	Age	Sex	Signs and Symptoms	Location	Imaging	Resection	RT	IHC/Molecular Markers (+)	Symptom Control	Follow-up (Months)	Recurrence
Wang et al. [6]	2022	56	M	Paraparesis. Bilateral pain, temperature, and touch hypoesthesia below inguinal level.	T10–T12	MRI: T1 hyperintense and T2 hypointense. Uncertain enhancement.	NT	No	HMB-45, MelanA Ki-67 = 5%	Worsened	17	No
Kim et al. [7]	2021	78	M	Right hemiparesis with numbness. Gait disturbance.	C3–C5	CT: hyperdense mass with heterogeneous core; MRI: T1 hyperintense, T2 hypointense. Mild enhancement.	T	No	MelanA, S-100, CD-68. Ki-67 < 5%.	-	-	-
Dubey et al. [8]	2018	35	F	Cervicothoracic back pain. Spastic paraparesis.	C6–T6	MRI: T1 hyperintense, T2 hypointense. Syrinx formation cranial and caudal. Heterogeneous enhancement.	NT	No	-	Improved	6	No
Wagner et al. [9]	2015	63	M	Right progressive hemiparesis. Bilateral positive Babinski sign.	C2–C3	MRI: T1 hyperintense, T2 isointense. No diffusion restriction.	T	Yes	S-100, MelanA, HMB-45.	Improved	18	Yes
Muthappan et al. [10]	2012	61	F	Right spastic hemiparesis and paresthesia. Loss of fine motor skills. Right hyperreflexia with positive Hoffman and Babinski signs.	C3–C4	MRI: T2 hyperintense.	T	No	S-100, MelanA, HMB-45. Ki-67 < 2%.	Stable	36	No
Eskandari et al. [11]	2010	45	M	Paraparesis with paresthesia and pain, worse on the right. Bowel and bladder urgency. LE hyperreflexia.	T11	MRI: T1 hyperintense, T2 hypointense with associated syrinx.	ST	Yes	-	Stable	36	Yes
Perrini et al. [1]	2009	79	F	Paraparesis with paresthesia. Urinary sphincter dysfunction. Hyperreflexia and myelopathy.	T10–T11	MRI: T1 hyperintense, T2 hypointense. Homogeneous enhancement.	ST	No	S-100, MelanA, HMB-45	Improved	30	Yes
Caruso et al. [12]	2009	62	M	Progressive paraparesis, spastic gait, hyperreflexia in both legs, and clonus on the left. Bilateral positive Babinski sign. Touch and pain hypoesthesia at T11 level.	T11–T12	MRI: T1 hyperintense, T2 hypointense.	T	No	S-100, HMB-45. Ki-67 < 2%.	Improved	24	No
Karikari et al. [5]	2009	20	M	Right LE paresis.	T12	MRI: T2 hyperintense. Homogeneous enhancement.	T	No	S-100, HMB-45, MART-1.	-	1.5	No
		32	F	Bilateral LE paresthesia. Patchy sensory loss on the left foot.	T10	MRI: T1 hyperintense. Homogeneous enhancement.	T	No	S-100, MART-1.	Resolved	3	No
Chacko et al. [13]	2008	22	M	Spastic paraparesis, bowel and bladder dysfunction. Pain, touch, and temperature hypoesthesia below T6. Reduced lower limbs proprioception. Hyperreflexia.	T6–T11	MRI: T2 hypointense. Homogeneous enhancement. Associated syrinx.	T	No	S-100, HMB-45, vimentin.	Improved	96	No
Horn et al. [14]	2008	37	F	Progressive paresthesia and dysesthesias of bilateral UE and LE.	C1–C3	MRI: T1 hyperintense, T2 hypointense. Homogeneous enhancement.	T	No	MelanA	Stable	38	Yes
		37	F	Thoracic back and left LE pain.	T9–T10	MRI: T1 hyperintense, T2 isointense. Homogeneous enhancement.	T	No	S-100, MelanA	Improved	16	Yes
		48	M	Paraparesis, bilateral LE paresthesia and urinary incontinence. Hyperreflexia with myelopathic signs.	T12	MRI: T2 hypointense. Homogeneous enhancement.	T	No	S-100	Improved before recurrence.	185	Yes
Turhan et al. [15]	2004	19	F	Lumbar pain. Paraparesis with hyperreflexia. Hypoesthesia below T10.	T8	MRI: T1 hyperintense, T2 hypointense. Associated syrinx. Homogeneous enhancement.	T	No	S-100, HMB-45. Ki-67 < 1%.	-	36	No

Abbreviations: IHC; immunohistochemistry. LE; lower extremity. T; gross total resection. NT; near total resection. ST; subtotal resection. RT; radiotherapy. UE; upper extremity. Outcome: W; worsened, I; improved, S; stable.

## Data Availability

No new data were created or analyzed in this study.
